# Recurrent Sporadic Bilateral Retinoblastoma in a Child with 13q Deletion Syndrome

**DOI:** 10.7759/cureus.6618

**Published:** 2020-01-10

**Authors:** Laila Tul Qadar, Syed Ali Shazif Baqari, Hira Maab, Sarrah Ali Asghar, Muhammad Saad Hafeez

**Affiliations:** 1 Internal Medicine, Dow University of Health Sciences, Karachi, PAK; 2 Pediatric Oncology, Shaukat Khanum Memorial Cancer Hospital and Research Center, Lahore, PAK; 3 Surgery, Aga Khan University Hospital, Karachi, PAK

**Keywords:** retinoblastoma, enucleation, 13q deletion syndrome, intraocular tumors, intravenous chemotherapy

## Abstract

13q syndrome is a chromosomal abnormality in which there is a pathognomic deletion of the genetic material on the long arm (q) of chromosome 13. Phenotypes of this syndrome are variable depending on the location of the deleted segment. The main manifestations of the syndrome include mental retardation, craniofacial dysmorphism, and increased susceptibility to tumors. We report a unique case of recurrent sporadic bilateral retinoblastoma (Rb) in a four-year-old boy carrying 13q (q12q14) interstitial deletion, which was treated successfully via enucleation and chemotherapy. Where most patients with familial Rb receive a single mutated Rb1 allele as the ‘first hit’, a small number of patients encounter interstitial deletion of the long arm of chromosome 13, resulting in the loss of the tumor suppressor Rb1 gene and presenting as sporadic cases.

## Introduction

Retinoblastoma (Rb) is one of the most common intraocular pediatric malignancies; however, it remains a rare form of childhood cancers, accounting for only 4% of the total [1]. The prevalence of Rb is one in every 15,000 to 18,000 live births [2]. This disease presents in infancy or early childhood, with the majority of cases diagnosed before the age of four years [3]. It rarely manifests in older children or adults. The pathogenesis of Rb primarily involves the conversion of a normal retinal cell into its malignant prototype as a result of biallelic (two-hit) inactivation of the Rb1 gene. Both heritable and nonheritable variants of the disease exist. In its heritable form, one hit is genetically present; conversely, in somatic disease, both hits are obtained during retinal development, causing Rb [1]. Rb manifests unilaterally in approximately two-thirds of all patients and bilaterally in one-third of the total [3]. The most frequent presenting sign of Rb is leukocoria which is a whitish pupillary reflex [1].

13q deletion syndrome is genetically characterized by the obliteration of one segment of the long arm (q) on chromosome 13q14. Clinical symptoms in 13q deletion syndrome are concurrent with the functions of the lost genes present in that segment; these include eye tumors (Rb), variable degrees of mental impairment, and characteristic facial features, including high forehead, prominent philtrum, and anteverted earlobes which are more common in male children [4]. We report a case of recurrent sporadic bilateral Rb with concomitant 13q deletion syndrome in a four-year-old boy. The first presentation of bilateral Rb was evident in the patient when he was three years old, for which he was administered intravenous chemotherapy.

## Case presentation

A three-year-old boy was referred to Shaukat Khanum pediatric oncology ward in August 2017, with a five-day history of leukocoria in the left eye (LE). On inspection, he looked malnourished and developmentally delayed with evident dysmorphic facial features like high forehead, prominent philtrum, and anteverted earlobes. He had an unremarkable family history. On ophthalmic examination of the right eye (RE), there was no hyperemia or pain; however, a swelling was noticed which presented with mild changes in the eye contours. Later on, examination under anesthesia (EUA) concluded that the patient had grade D Rb in his LE and grade C Rb in his RE (as per International Classification for Intraocular Retinoblastoma). 

Initial management was purely chemotherapy. Six cycles of intravenous VEC (carboplatin, vincristine, and etoposide) chemotherapy were started. RE was better responding to the therapy than the LE on EUA. Three cycles of chemotherapy were further given including two laser therapy sessions. After the eighth cycle, the patient presented with pneumonia for which antibiotics had to be administered.

**Figure 1:**
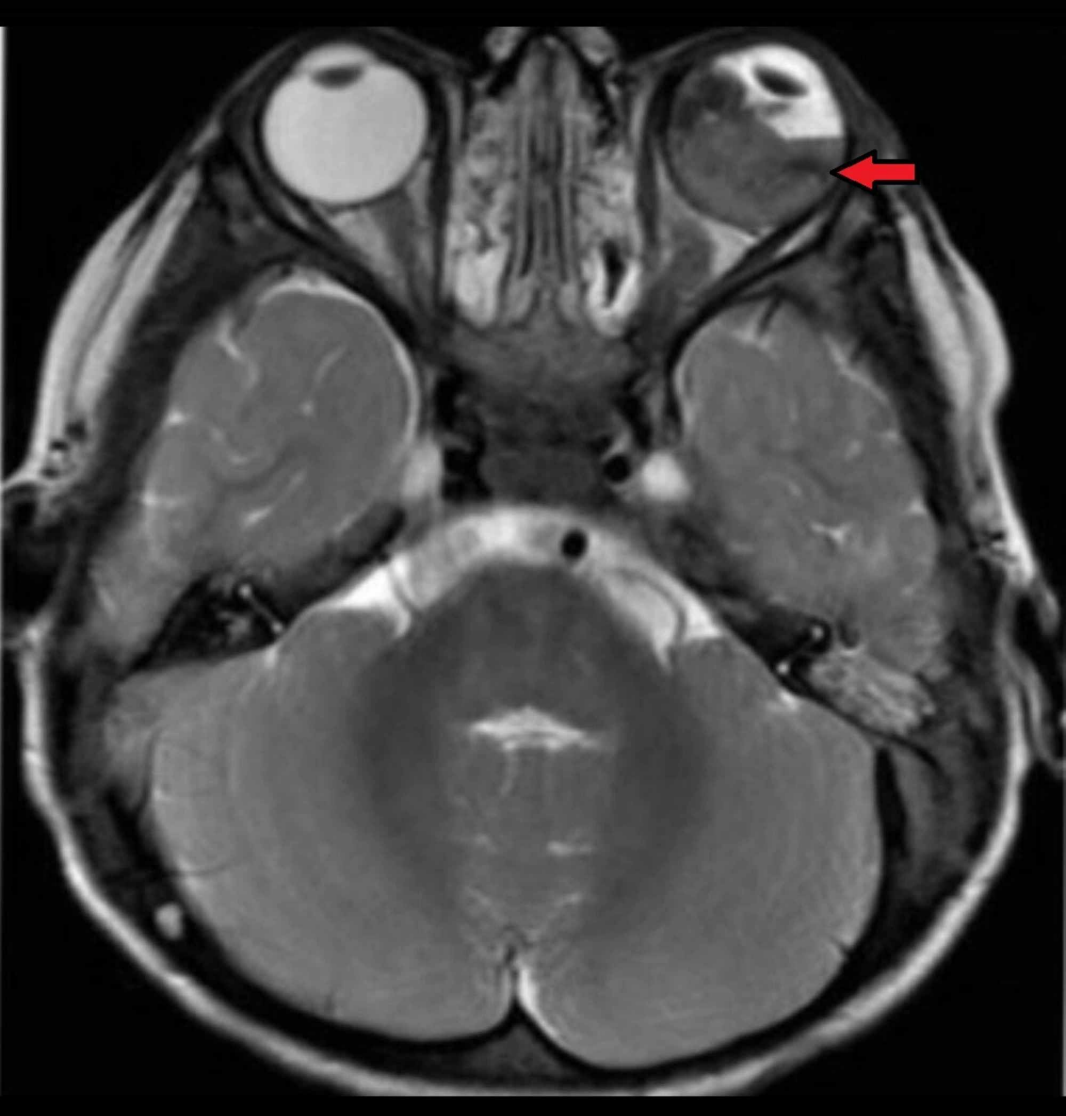
Axial T2-weighted MRI of the brain and orbit showing retinoblastoma in left eye along with retinal detachment (red arrow)

The patient was discharged after one year of complete chemotherapy. He returned three months post-treatment, with the complaint of leukocoria in LE along with massive proptosis. LE showed retinal detachment on magnetic resonance imaging (Figure 1).

**Figure 2:**
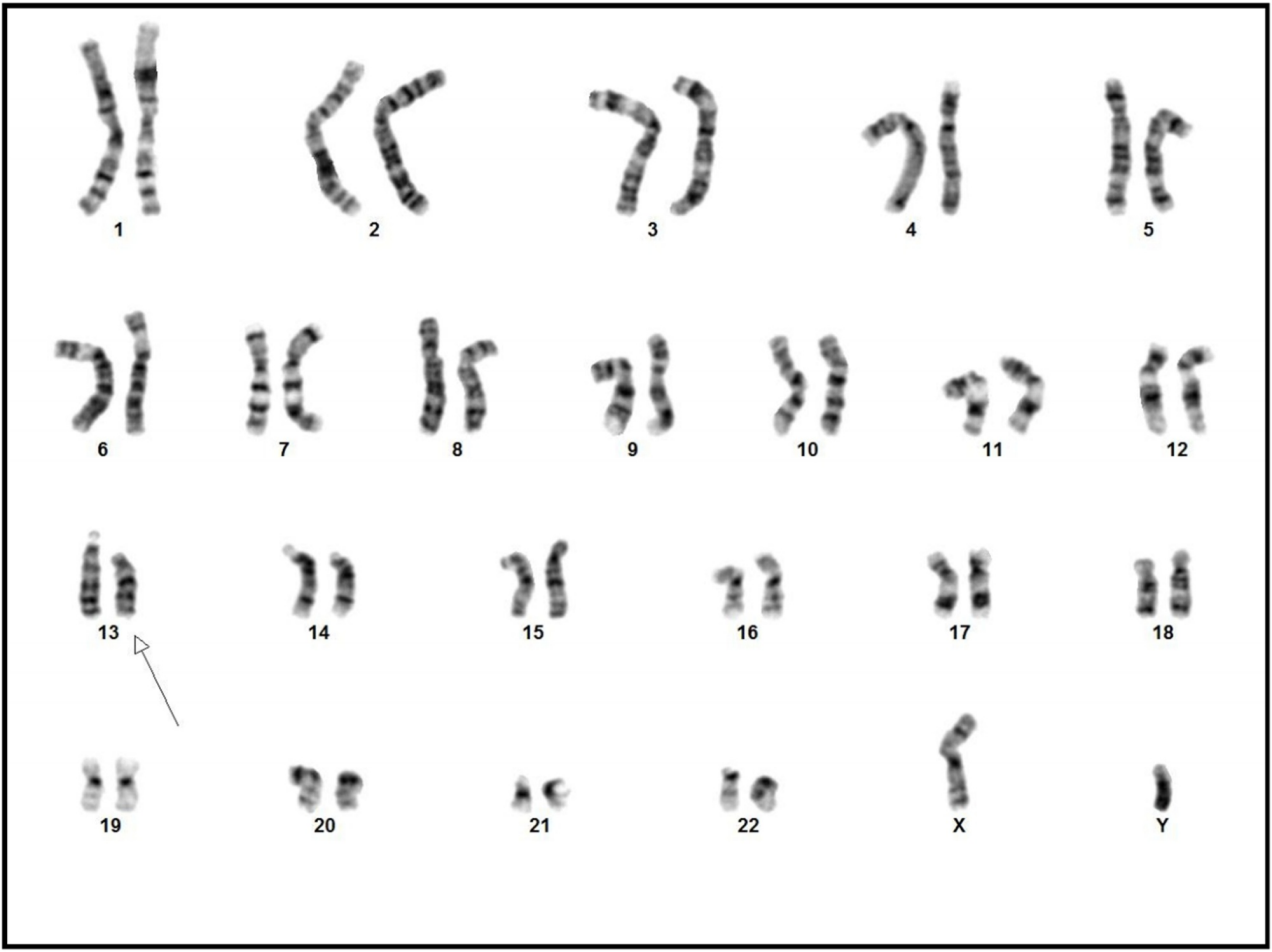
Chromosomal analysis revealing 46XY karyotype with 13q deletion

Intravitreal methotrexate was administered to the patient. Later, he was prepared for laser and six cycles of IVAD (vincristine, doxorubicin, ifosfamide), out of which only four cycles could be infused because of the side effects. RE disease had already regressed. LE, on the other hand, was deteriorating due to chemotoxicity and showed poor response. It was finally decided to enucleate the LE. The histopathological examination of the enucleated LE revealed moderately differentiated RB occupying the entire vitreous cavity with massive choroidal invasion and without optic nerve invasion. After the enucleation, a prosthetic eye was implanted in the left orbit. To investigate the genetic causes of recurrent RB, cytogenic analysis was carried out in which 13q deletion (q12q14) was detected (Figure 2). 

The results established that the patient had 13q deletion syndrome which showed male chromosome complement in which all 20 GTG banded cells had an interstitial deletion of chromosome 13q, and no normal 46XY cells were present.

After the LE enucleation, four-weekly EUA follow-up was advised, which was later reduced to three monthly visits. The patient has been coming for regular check-ups since seven months after the treatment, but no recurrence has been reported as yet.

## Discussion

Rb is divided into unilateral Rb (involving one eye) and bilateral Rb (involving both eyes) according to its laterality. Another approach to the classification of Rb is sporadic (90% cases) and familial (in which the patient inherits the condition from one of his parents-10 % of all cases) [5].

Our patient is a case of bilateral Rb with no history of affected parents; this makes his condition ‘sporadic’ at the clinical level. This means that the disease is inherited from apparently healthy parents. In Pakistan, bilateral Rb is comparatively more prevalent than its unilateral counterpart. In Pakistan, the mean age at presentation of bilateral Rb is 31.10 months as compared to unilateral Rb whose mean age of diagnosis is 38.97 months. A geographical variation is evident in Rb prevalence as in India, the neighboring country, the reported age of diagnosis is quite early (23.98 months) [6].

Only rarely, sporadic bilateral Rb occurs in a setting of interstitial deletion of the long arm(q) of chromosome 13 (commonly known as the 13q deletion syndrome); our case was one such occurrence. Cytogenetic analysis along with the banding technique is conclusive in finding the exact location and pattern of deletion in chromosome 13. 13q deletion syndrome does not always involve the exact same location in the long arm; it varies between proximal segment deletion and interstitial deletion. Our patient’s cytogenetic analysis revealed the classic interstitial deletion of 13q. This chromosomal zone has one or more genes that are responsible for normal retinal development. We, however, cannot predict for sure that the sporadic Rb manifested only as a consequence of 13q deletion. This tumor has a number of different causes. Further workup is needed to determine the exact specificity of lost genetic material to put Rb under the radar of the consequences of 13q deletion syndrome [7].

Interstitial 13q deletion syndrome is associated with peculiar findings in terms of growth, facial appearance, and intellect. Dysmorphic facial features are evident in these patients, as in our case. Broad nasal bridge, short nose with a broad tip, distinct external ears, and muscular hypotonia are some of the apparent clinical findings. Other associations with the syndrome include gastrointestinal tract abnormalities, Rb, and failure to thrive [8].

Rb management is complex. It involves acquiring complete information on the tumor size, its invasion to the orbit structures, and its proximity to surrounding vital orbital components such as optic disc, choroid, and sclera. The patient has to undergo a series of investigations before the treatment of choice is decided. Nowadays, the preferred treatment for bilateral Rb is intravenous (IV) chemoreduction which aims to treat both eyes as well to diminish the proliferative risk of intracranial tumors (pinealoblastoma; also called trilateral Rb) [9]. The efficacy of IV chemoreduction has been proven by studies in which the anticipated risk of pinealoblastoma was reduced to nil after the administration of the respective neoadjuvant therapy [9]. The standard three-agent chemotherapy protocol is practiced most commonly in most Rb centers. The same protocol was appreciated initially in our case as well.

Extensive Rb is approached via enucleation. Enucleation often forms the last-resort treatment in Rb cases. In this patient, the LE showed poor response to chemotherapy and instead of the tumor subsiding, the eye condition was deteriorating and it could not be salvaged; thus, enucleation was decided for the LE. This process involves cautious removal of the eye and an optic nerve segment followed by implantation of a prosthesis which is held intact via suturing to the four rectus muscles [10]. Lately, in Pakistan, an elevating incidence of enucleation and mortality secondary to Rb has been observed, and this has been attributed to aberrant diagnosis, detained referrals, advanced disease at presentation, lack of access to advanced medical facilities, poor compliance of medical advice, illiteracy, poor economic conditions, and nonexistence of standard management protocols [1]. If primary knowledge of distinguishing ‘a whitish pupillary reflex’ from a normal pupil is present in general masses, the patient can be brought to the hospitals with an earlier, curable form of the disease.

## Conclusions

Primary care providers at the district level in our set-up should also be trained to recognize such oncologic cases earlier and initiate timely referrals. Health care professionals especially ophthalmologists must be aware that consecutive Rb with concomitant mental impairment puts 13q deletion syndrome on the topmost differential diagnosis and hence urgent karyotyping must be performed to confirm it. Routine eye check-ups in children must be made fundamental at least for the first five years of life. A simple finding such as leukocoria is vital for detecting the disease and thus should never be ignored.

## References

[1] Ortiz MV, Dunkel IJ (2016). Retinoblastoma. J Child Neurol.

[2] Rao R, Honavar SG (2017). Retinoblastoma. Indian J Pediatr.

[3] Broaddus E, Topham A, Singh AD (2009). Incidence of retinoblastoma in the USA: 1975-2004. Br J Ophthalmol.

[4] Mitter D, Ullmann R, Muradyan A (2011). Genotype-phenotype correlations in patients with retinoblastoma and interstitial 13q deletions. Eur J Hum Genet.

[5] Pichi F, Lembo A, De Luca M, Hadjistilianou T, Nucci P (2013). Bilateral retinoblastoma: clinical presentation, management and treatment. Int Ophthalmol.

[6] Adhi MI, Kashif S, Muhammed K, Siyal N (2018). Clinical pattern of retinoblastoma in Pakistani population: review of 403 eyes in 295 patients. J Pak Med Assoc.

[7] Soliman SE, Racher H, Zhang C, MacDonald H, Gallie BL (2017). Genetics and molecular diagnostics in retinoblastoma--an update. Asia Pac J Ophthalmol (Phila).

[8] Brennan RC, Qaddoumi I, Billups CA, Kaluzny T, Furman WL, Wilson MW (2016). Patients with retinoblastoma and chromosome 13q deletions have increased chemotherapy-related toxicities. Pediatr Blood Cancer.

[9] Shields CL, Meadows AT, Shields JA, Carvalho C, Smith AF (2001). Chemoreductionfor retinoblastoma may prevent intracranial neuroblastic malignancy (trilateral retinoblastoma). Arch Ophthalmol.

[10] Zigiotti GL, Cavarretta S, Morara M (2012). Standard enucleation with aluminium oxide implant (bioceramic) covered with patient’s sclera. ScientificWorldJournal.

